# Metastatic mediastinal malignant tumors of gastrointestinal origin with occult primary lesions: a case report

**DOI:** 10.3389/fonc.2025.1625668

**Published:** 2025-07-09

**Authors:** Yaxuan Liu, Liang liang Yang, Wen teng Hu, Rui jiang Lin, Song la Bai, Min jie Ma, Biao Han

**Affiliations:** ^1^ Department of Thoracic Surgery, The First Hospital of Lanzhou University, Lanzhou University, Lanzhou, China; ^2^ The First Clinical Medical College of Lanzhou University, Lanzhou University, Lanzhou, China; ^3^ The International Science and Technology Cooperation Base for Development and Application of Key Technologies in Thoracic Surgery, Lanzhou, China; ^4^ Control Center of Thoracic Surgery of Gansu Province, The First Hospital of Lanzhou University, Lanzhou, China

**Keywords:** cancer of unknown primary, mediastinal metastasis, gastrointestinal immunophenotype, solitary metastasis, diagnostic biomarkers, liquid biopsy

## Abstract

Cancer of unknown primary origin (CUP), accounting for 3–5% of malignancies, poses significant diagnostic challenges because of the absence of identifiable primary lesions. While common occult primary tumors involve the lung or pancreas, gastrointestinal (GI)-originated mediastinal metastases are exceedingly rare. A 54-year-old male presented with chest tightness and dyspnea. Imaging revealed a 45.5 × 36.3 mm anterior mediastinal mass. Pathological evaluation postresection revealed metastatic moderately differentiated adenocarcinoma with immunohistochemical (IHC) features (CK20+/Villin+/CK7−/TTF-1−) suggestive of GI origin. Despite comprehensive evaluations (gastroscopy, PET-CT), no primary lesions were detected. Chronic atrophic gastritis (C2) was noted, but malignancy was excluded. This case underscores the diagnostic complexity of GI-profile mediastinal CUP and highlights limitations in conventional imaging. Molecular profiling (e.g., *KRAS/NRAS/BRAF* mutation) and advanced diagnostics (ctDNA analysis) are critical for accurate classification and tailored therapy. Long-term surveillance remains essential, as 12% of CUPs reveal primaries during follow-up.

## 1 Introduction

Cancer of unknown primary (CUP) is a malignant tumor that is pathologically identified and diagnosed by the presence of a metastatic malignancy in which the primary lesion cannot be identified. CUPs account for approximately 3–5% of all malignancies. CUP primary lesions are most commonly detected in the lung and pancreas, followed by other gastrointestinal and gynecologic malignancies, and in some patients, further autopsy fails to identify the origin (1). Direct invasion or lymph node metastasis (especially small cell carcinoma) accounts for approximately 60–70% of tumors that metastasize to the mediastinum most frequently (2) and are rarely seen in gastrointestinal metastases. Here, we report a case of metastatic mediastinal malignancy with an insidious primary lesion (occult primary tumor) in which the site of origin was still not identified after detailed gastrointestinal evaluation, return visits for pathological immunohistochemical findings, and PET–CT examination; thus, such a case is even rarer.

## 2 Case presentation

**Figure 1:**
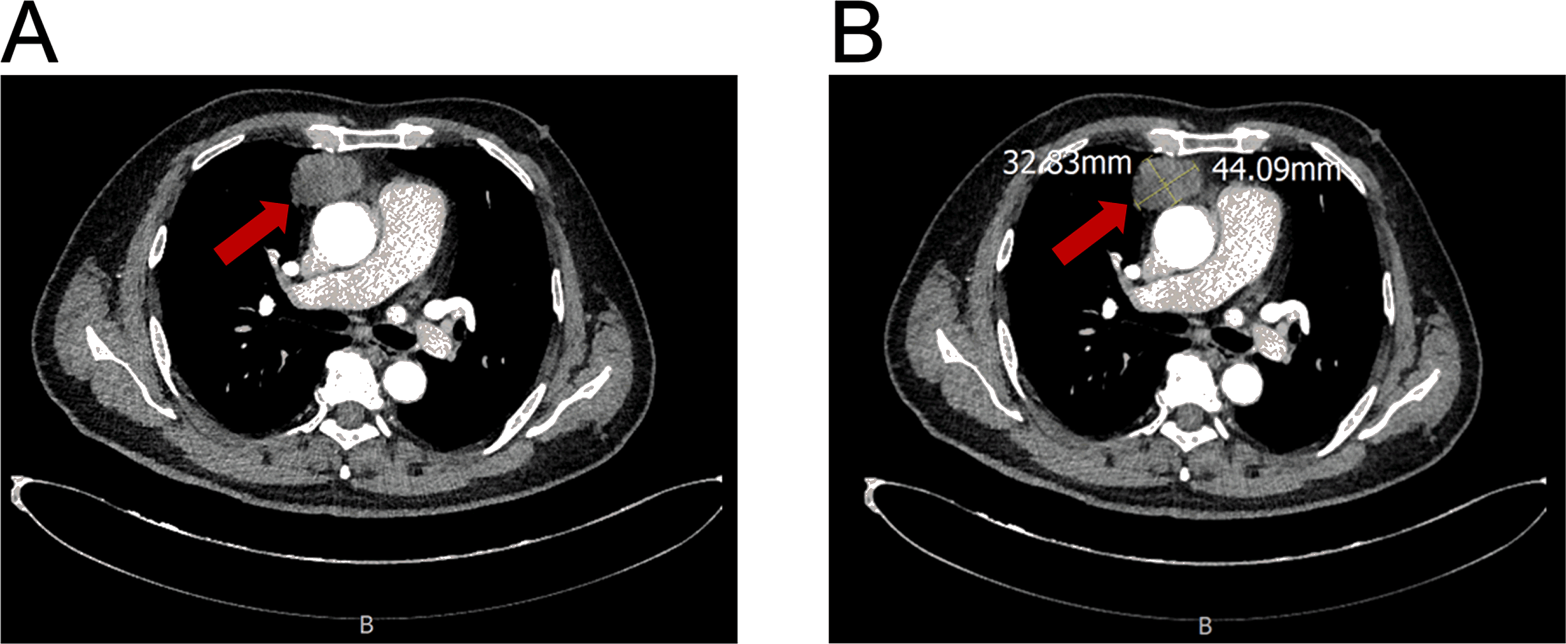
Contrast-enhanced CT of an anterior mediastinal mass. **(A)** Axial image showing the mass (arrow). **(B)** Image demonstrating tumor measurement (44.09 mm x 32.63 mm).

**Figure 2:**
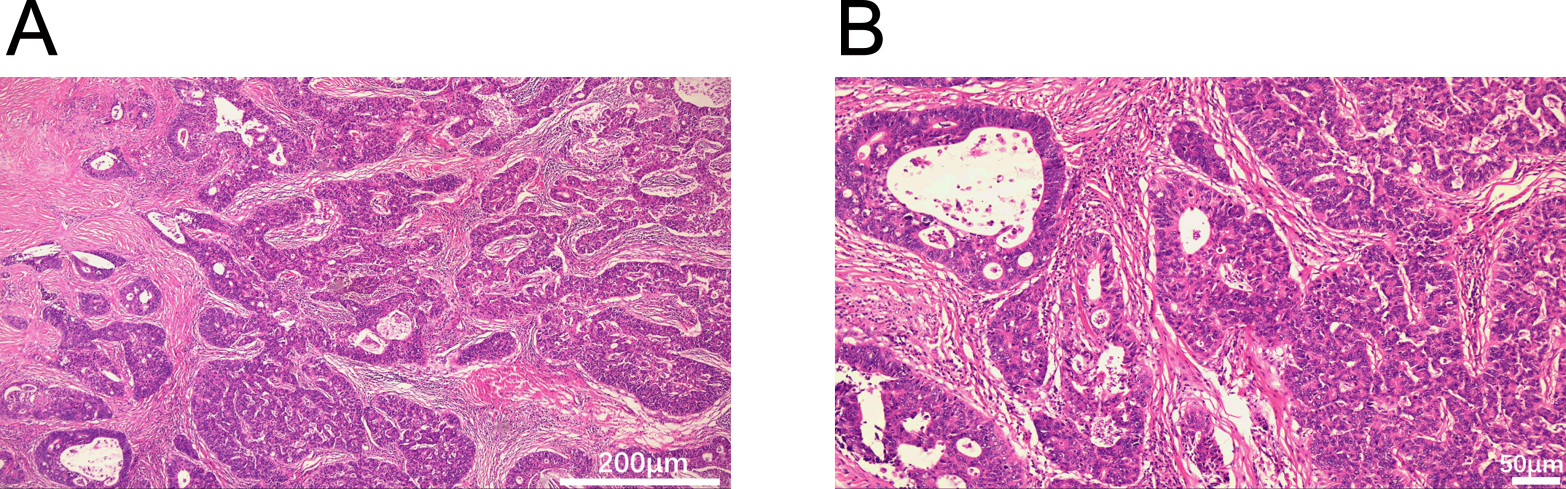
Histopathological features of the mediastinal mass (H&E staining). **(A)** Glandular architecture (40×). **(B)** Glandular architecture (100×). Scale bars: 200 μm **(A)**, 50 μm **(B)**.

**Figure 3:**
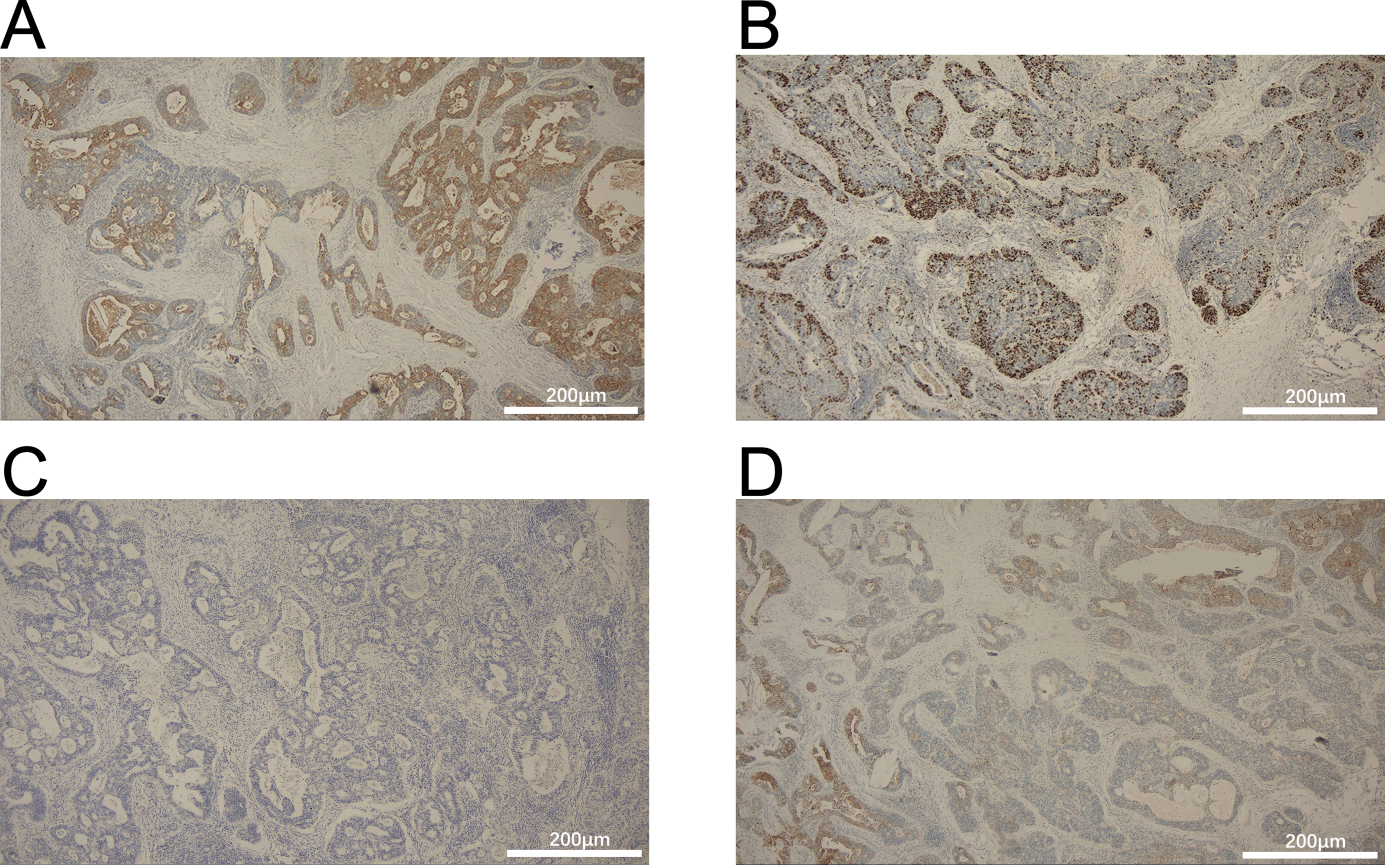
Immunohistochemistry for **(A)** CK20, **(B)** villin (+), **(C)** thyroid transcription factor-1 (TTF-1), and **(D)** Ki-67. Scale bars: 200μm.

A 54-year-old man presented with chest tightness and shortness of breath for 1 month and was found to have a space-occupying lesion in the right anterosuperior mediastinum on examination, and he had intermittent alcohol consumption and no history of smoking. He had a history of hypertension for more than 10 years. The patient’s hypertension was well controlled (150/80 mmHg) with nifedipine and betaloc, with no surgical contraindications. Imaging examination revealed soft tissue shadows of approximately 44.09 mm × 32.83 mm in size in the anterior mediastinum (**Figure 1**), and CT values of approximately 48 U and 75 U were measured in the enhanced two phases; abdominal color ultrasound revealed no significant abnormalities in the liver, gallbladder, pancreas, spleen, or both kidneys. No significant abnormalities were found in blood tests after admission; stool routine tests: routine microscopy of stool revealed; 0 RBC/HPF, 0 WBC/HPF, and occult blood tests were negative. After the preoperative examination was complete, no surgical contraindications were found. Thoracoscopic resection of mediastinal lesions was performed under general anesthesia with endobronchial intubation. Thoracic exploration revealed no pleural effusion; mediastinal lesions were located in the anterior mediastinum, with an intact capsule that was approximately 50 mm × 40 mm in size and hard. The following procedures were used for mediastinal tumor resection: extracapsular free mediastinal tumor, ligation of nutrient vessels, removal of adipose tissue around the tumor, and complete resection of the mediastinal tumor. Postoperative histopathology revealed fibrous hyperplasia with atypical cells arranged in irregular glandular tubular, acinar and papillary patterns and infiltrative growth (**Figure 2**). Immunohistochemistry: A1 block: CK20 (+); Villin (+); CD5 (-); CD117 (-); CD56 (-); CK (CAM5.2) (+); CK7 (-); EMA (+); Ki-67 (40%); Napsin A (-); p40 (-); p53 (80%); p63 (-); TTF-1 (-); CK8amp18 (+) (**Figure 3**). On the basis of morphologic and immunohistochemical findings suggestive of moderately differentiated adenocarcinoma, these findings suggest that the gastrointestinal tract may be the predominant site. Therefore, gastroscopy was performed on the 7th postoperative day, which revealed that the overall gastric mucosa was swollen and that the gastric mucosa was diffusely red. The structure of the gastric area was thick and reticular, with multiple punctate congestion erythema, increased secretions, white turbidity, and RAC not visible. The mucosa from the antrum to the gastric angle and lesser curvature of the gastric body was red and white, was mainly white, and could penetrate the submucosal vascular network. Tips: atrophic gastritis (C2) and no obvious space-occupying lesions, ruling out obvious evidence of a malignant tumor in the stomach. Because the patient could not tolerate compound polyethylene glycol, colonoscopy was not performed. PET–CT revealed no significant abnormalities in the digestive tract. Later, we advised the patient to have the pathological section of the postoperative sample removed and consult further pathological results at the Second Hospital of Lanzhou University. Pathological findings revealed adenocarcinoma (mediastinal mass), carcinoma, 3 lymph nodes and no metastasis. CK20 (+); EMA (+); CK8/18 (+); CK (CAM5.2) (+); Villin (+); CD56 (−); CD5 (−); CD117 (−); TTF-1 (−); napsin A (−); P53 (mutant); P63 (−); P40 (−); CK7 (−); Ki67-positive cells were 30%; and finally, CUP was diagnosed after multidisciplinary discussion at our hospital (MDT).

## 3 Discussion

In this case, the primary tumor was not identified after extensive gastrointestinal evaluation and PET-CT, highlighting the diagnostic challenges of occult primary malignancies, and emphasizing the role of molecular classification analysis in guiding management. CUP accounts for 3-5% of all malignancies and CUP is defined as: metastatic disease present despite thorough diagnostic evaluation but no identifiable primary site (3). Although the most common occult primary is lung and pancreatic cancer, followed by gastrointestinal (GI) and gynecologic malignancies (1), the present case highlights the unusual presentation of metastatic mediastinal adenocarcinoma with strong immunohistochemical (IHC) evidence of gastrointestinal origin, but no identifiable primary lesion — a condition that is hardly reported in the literature. Patients’ mediastinal malignancies exhibited morphologic and IHC features consistent with metastatic adenocarcinoma (CK20 +/Villin +/CK7 −/TTF-1 −), a feature often associated with gastrointestinal malignancies, particularly colorectal or gastric primary tumors (4). However, upper GI endoscopy showed only chronic atrophic gastritis (C2) without neoplastic lesions and PET-CT showed no hypermetabolic lesions in the GI tract. This discordance highlights two key challenges in CUP management: the limitations of conventional imaging in detecting occult primary and the possibility that microscopic or regressing lesions may escape detection. Notably, Gollub et al. reported that FDG-PET has 49% sensitivity for detecting advanced adenomas (5), whereas early mucosal lesions (e.g., signet ring cell carcinoma) may lack FDG avidity, F-FDG PET/CT has low sensitivity for signet ring cell gastric cancer. The FDG-PET based detectability of this tumor ranged from 14.3% to 70.6%, which was significantly lower than that of non-signet ring cell carcinoma (52.9-100%) and consistent with its significantly lower SUV (6). The limited sensitivity of FDG-PET in detecting early GI lesions (14.3–70.6% in our case) aligns with prior reports on signet-ring cell carcinomas (5). This underscores the critical importance of auxiliary tools such as ctDNA analysis. When integrated with genomic analysis, ctDNA analysis is capable of identifying potential primary lesions or mutations associated with targeted therapies in approximately 80% of cases (7). The rarity of metastatic malignant mediastinal tumors of gastrointestinal origin further complicates the diagnosis. While lung and breast cancers predominate in mediastinal metastases (60–70%) (2), gastrointestinal tumors metastasizing to the mediastinum are extremely rare (< 5%) (8). One included 8,491 patients with colorectal cancer, and only 0.7% presented with mediastinal metastases (9). Our case is consistent with these statistics, but increases in complexity as there are no other metastatic sites, which at the same time suggests a unique biological behavior. The uniqueness of this biological behavior is characterized by the concurrent rarity of its occurrence site (the mediastinum), the isolated nature of the metastatic lesion, and the profound concealment of the primary lesion, which deviates from the expected typical metastatic patterns of gastrointestinal cancer. Consequently, we propose that this phenomenon represents a “distinct biological behavior.” This distinctiveness holds substantial significance for comprehending the complexity of tumor metastasis, refining diagnostic strategies for CUP (such as prioritizing molecular profiling and ctDNA analysis), and investigating potential personalized treatment options.

IHC profiles (CK20 +/Villin +/p53 overexpression) strongly support GI origin. p53 mutations are present in 80% of colorectal cancers and 50% of gastric adenocarcinomas (10) and may further narrow the differential diagnosis if SATB2 is negative. In this context, future assessments should prioritize extended IHC panels (e.g., *SATB2, HER2*) and molecular analyses (e.g., *KRAS/NRAS/BRAF* mutations) to guide source-specific therapy (11).

Clinically, this case highlights the need for multidisciplinary escalation when standard diagnosis fails. Endoscopic ultrasonography (EUS) guided biopsy, capsule endoscopy and even abdominal exploration may detect occult primary lesions. Empirical therapies based on IHC analysis (eg, FOLFOX suspected of colorectal origin) remain controversial, but there are retrospective data supporting that show improved survival in CUPs of the GI spectrum (12).

In conclusion, this case illustrates the diagnostic mystery of mediastinal CUPs in GI contours and highlights critical gaps in current diagnostic modalities. It highlights the importance of integrating advanced molecular diagnostics (eg, ctDNA analysis) and long-term surveillance, and Hemminki K et al. report that approximately 12% of CUP patients have a confirmed primary site on follow-up based on Swedish Cancer Registry data (n = 11, 856) (13).

## 4 Conclusion

This report characterizes an unusual presentation of a solitary anterior mediastinal metastasis demonstrating definitive gastrointestinal immunohistochemical features (CK20+/Villin+/CK7−/TTF-1−), wherein comprehensive diagnostic assessment failed to identify a primary lesion. This case underscores four critical clinical insights: (i) mediastinal metastases exhibiting gastrointestinal immunophenotype constitute a diagnostically challenging subset of cancer of unknown primary (CUP), particularly when manifesting as isolated lesions; (ii) the confirmed metastatic presentation with occult primary necessitates molecular profiling (KRAS/NRAS/BRAF genotyping and ctDNA analysis) to guide tissue-of-origin classification; (iii) sustained surveillance is warranted, given that approximately 12% of CUPs reveal primaries during follow-up; and (iv) multidisciplinary therapeutic strategy formulation. This presentation exemplifies diagnostic complexities in metastatic disease biology and reinforces the necessity of integrating advanced diagnostics into CUP evaluation paradigms.

## Data Availability

The original contributions presented in the study are included in the article/supplementary material. Further inquiries can be directed to the corresponding authors.
